# Manganese handling in plants: Advances in the mechanistic and functional understanding of transport pathways

**DOI:** 10.1017/qpb.2025.10012

**Published:** 2025-06-20

**Authors:** Bastian Meier, Oriana Mariani, Edgar Peiter

**Affiliations:** Plant Nutrition Laboratory, Institute of Agricultural and Nutritional Sciences, Martin Luther University Halle-Wittenberg, Halle (Saale), Germany

**Keywords:** intracellular distribution, manganese transport, manganese uptake, micronutrients, post-translational regulation

## Abstract

As the catalytic centre of the oxygen-evolving complex in photosystem II and a co-factor of glycosyltransferases and many other proteins, manganese (Mn) is essential for plants and a limiting factor for crop production. However, an excessive Mn availability is toxic to plants. Therefore, mechanisms need to be in place to maintain Mn homeostasis under fluctuating Mn availability. This review summarises our current understanding of the mechanisms that move Mn from the soil to its cellular targets and maintain Mn homeostasis. We zoom in from the whole-plant perspective to the intracellular allocation of the metal by transport proteins of different families acting in concert. In particular, organellar Mn supply by members of the recently identified bivalent cation transporter family and the post-translational regulation of Mn transporters by calcium-regulated phosphorylation have been a focus of current research. Finally, the emergent diversity of Mn handling beyond the Arabidopsis model will be addressed.

## Graphical abstract

**Figure:**
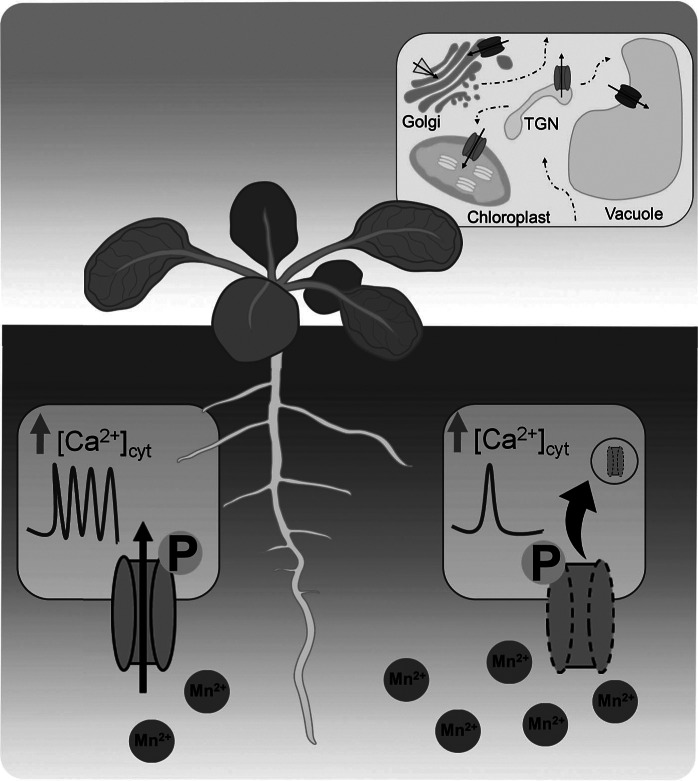

## 1. Introduction

Manganese (Mn) is an essential trace metal that participates in numerous physiological processes and is indispensable for plant growth and development (Peiter, 2014). Hence, Mn can be a limiting factor for crop performance and yield formation (Assuncao et al., 2022). As a transition metal, Mn is integral to redox processes and electron transfer reactions (Lilay et al., 2024). It serves as a cofactor for various metalloenzymes, functioning as both a Lewis acid and redox catalyst. Owing to its role in the oxygen-evolving complex (OEC) in photosystem II, one of the earliest symptoms of Mn deficiency is a decline in photosynthesis, resulting from the reduced stability of the OEC and decreased electron transfer (Alejandro et al., 2023; Schmidt et al., 2016). Beyond photosynthesis, adequate Mn levels are crucial for a plethora of processes, including respiration, oxidative stress mitigation, glycosylation, matrix polysaccharide and lignin biosynthesis and phytohormone homeostasis (Andresen et al., 2018). These reactions take place in different cellular compartments, necessitating the distribution of the metal on various levels of scale, first between plant organs and subsequently between and within organelles (Bashir et al., 2021). Compared to other metals, such as iron (Fe) and zinc (Zn), the mechanisms of Mn sensing and transport are less well understood (Huang, Yamaji, & Ma, 2024). Since the transport and functions of Mn have last been addressed in comprehensive reviews (Alejandro et al., 2020; Andresen et al., 2018; He et al., 2021; Shao et al., 2017), we have witnessed a substantial progress in some areas of plant Mn biology. In particular, the Mn supply of organellar targets and the regulation of Mn transporters at the post-translational level has been the focus of recent research. It has also become apparent that plant species differ markedly in their Mn handling and their employment of orthologous transport proteins. This review focuses on new developments in Mn transport at the organismic and cellular levels, highlighting the importance of intracellular distribution and post-translational modifications.

## 2. Mn in soil and rhizosphere

Mn is acquired from the soil solution as Mn^2+^, whereas higher oxidation states, such as Mn(III) and Mn(IV), present in oxides, are unavailable to plants (Alejandro et al., 2020). Mn availability is thus primarily determined by soil chemistry, in particular pH and redox status, with acidic and reducing conditions increasing its availability. In particular, the soil’s redox potential may change rapidly and on a small scale, for example, upon drying and rewetting, demanding rapid adaptation of Mn uptake and handling. Additionally, H^+^ release by roots increases Mn availability in the rhizosphere by a factor of 100 per unit pH change. Exuded organic anions may chelate Mn and function as electron donors, further increasing Mn solubility. Conditions that provoke carboxylate exudation, such as phosphorus deficiency, thus lead to increased Mn availability and uptake (Lambers et al., 2015). Finally, microorganisms can mediate the redox cycling of Mn, and hence affect its availability to plants. Since Mn occurs in various oxidation states in soils and plants, we employ the generalised abbreviation Mn in this review when not referring to a specific oxidation state.

## 3. Getting in – pathways of Mn uptake

Prior to uptake into root cells, Mn may move apoplastically in the cortical cell walls towards the root endodermis. Coating of endodermal cells by a suberin layer physically prevents Mn influx in older root sections. Interestingly, deficiency of Mn, Fe and Zn led to delayed suberisation along the root axis (Barberon et al., 2016). This response depended on an intact ethylene signalling pathway, and likely serves to increase the absorptive area. Contrary to this finding in Arabidopsis, in roots of *Hordeum vulgare* (barley), the strength of Mn deficiency was decisive for endodermal suberisation, believed to either increase uptake into the xylem under mild deficiency or decrease leakage under severe deficiency (Chen et al., 2019).

**Figure 1.**
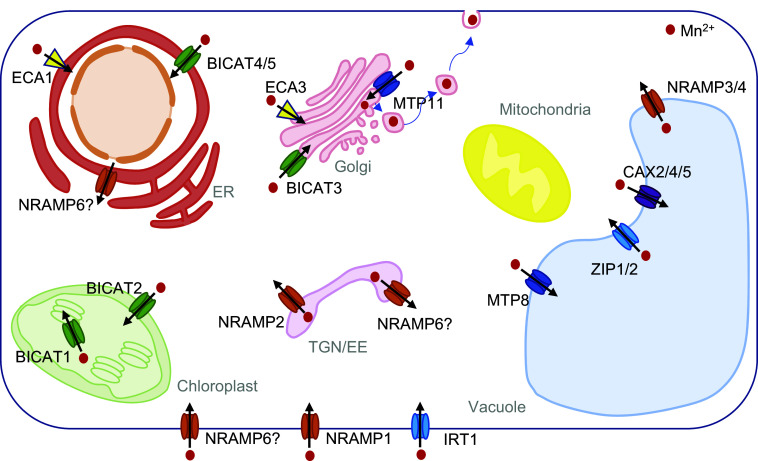
Schematic representation of a hypothetical *Arabidopsis thaliana* cell containing all transport proteins from various gene families with known localisation. These proteins include members of the bivalent cation transporter family (BICAT, green), metal tolerance protein family (MTP, dark blue), Zrt-Irt-like proteins (ZIP, light blue), natural resistance-associated macrophage proteins (NRAMP, orange), calcium exchangers (CAX, purple) and P_2A_-type ATPases (yellow). The MTP family is implicated in detoxification processes, with AtMTP11 facilitating Mn transport into the Golgi apparatus, followed by exocytosis (depicted by blue arrows). Under Fe-deficient conditions, AtMTP8 sequesters excess Mn into the vacuole. NRAMP family members mediate Mn import into the cytosol (AtNRAMP1/6) and from intracellular compartments, such as TGN/EE (AtNRAMP2), enhancing Mn accumulation in chloroplasts. AtNRAMP3/4 mediates Mn release from the vacuole. AtBICAT2/PAM71-HL/CMT1 facilitates Mn transport across the chloroplast envelope into the stroma, while AtBICAT1/PAM71 transfers Mn into the thylakoid lumen. AtBICAT3/PML3 channels Mn into the *trans-*Golgi cisternae, where it plays a crucial role in glycosylation and cell wall matrix polysaccharide synthesis. Other depicted transporters are described in Alejandro et al. (2020) and He et al. (2021).

A transporter of the natural resistance-associated macrophage protein (NRAMP) family, AtNRAMP1, is essential for the high-affinity uptake of Mn into Arabidopsis root cells (Cailliatte et al., 2010) (Figure 1). *In planta*, this protein also mediates the uptake of Fe with low affinity (Castaings et al., 2016), whereas high-affinity Fe uptake is pursued by iron-regulated transporter 1 (AtIRT1), a member of the Zrt/Irt-like family (Vert et al., 2002). Apart from Fe, AtIRT1 transports a range of other metal ions, including Mn (Korshunova et al., 1999). However, AtIRT1 does not contribute to Mn acquisition under conditions of sufficient Fe supply. After uptake, Mn may move symplastically from cell to cell via plasmodesmata towards the root vasculature and be released into the xylem apoplast, whereby the mechanism of xylem loading is still obscure.

Unlike AtIRT1 in Arabidopsis, the barley ortholog HvIRT1 is of high importance for the uptake and root-to-shoot translocation of Mn but is not involved in Fe acquisition (Long et al., 2018). In *Oryza sativa* (rice), the import of Mn into exo- and endodermal cells is primarily mediated by OsNRAMP5 (Sasaki et al., 2012), an ortholog of AtNRAMP1, as well as additional transporters of lower relevance, such as OsNRAMP1 (Chang et al., 2020). Release of Mn from those cells into the stellar apoplast is mediated by a transporter of the metal tolerance protein (MTP) family, OsMTP9 (Ueno et al., 2015). OsNRAMP5 and OsMTP9 are polarly localised at the distal and proximal side of the cells, respectively, thus forming a one-way road for Mn. Polar localisation has not yet been observed for AtNRAMP1.

## 4. Mn allocation, storage and sensing in the vasculature

Following its transfer into the root vascular system, Mn needs to be allocated to plant parts demanding the metal, while preventing critically high concentrations under high load. In rice, the OsNRAMP3 protein functions as molecular points in the node, distributing Mn to either young leaves and panicles or to old leaves, depending on the Mn status (Yamaji et al., 2013). This is brought about by OsNRAMP3-mediated loading of xylem transfer cells in enlarged vascular bundles as well as the subsequent loading of phloem parenchyma cells, also facilitated by OsNRAMP3. Under excessive Mn supply, OsNRAMP3 is degraded, allowing Mn to follow the main transpiration stream to old leaves. Xylem unloading of translocated Mn has recently been demonstrated to be a second task of OsNRAMP5 besides its function in root Mn uptake (Huang, Konishi, et al., 2024). In the sheath of rice leaves, this transporter is polarly localised in xylem parenchyma cells to supply Mn to this part of the leaf and restrict further translocation to the leaf blade.

Apart from OsNRAMP3 and 5, our picture of vascular Mn translocation in rice is sketchy, and it is even more so in Arabidopsis. Genes encoding several transport proteins permeating Mn are primarily or exclusively expressed in its vascular system, including AtNRAMP2 (Alejandro et al., 2017; Gao et al., 2018), AtNRAMP3 and 4 (Lanquar et al., 2005; Lanquar et al., 2010), AtNRAMP6 (Cailliatte et al., 2009; Li et al., 2022), yellow stripe like 1 (AtYSL1) and 3 (Waters et al., 2006), as well as the Ca^2+^/Mn^2+^-ATPase AtECA3 (Li et al., 2008). Deletion mutants in these proteins have Mn-related phenotypes, but their role in stellar Mn allocation is largely unclear, as the specific vascular cell types expressing the transporters have not been well identified. However, genetic interactions of some of the transporters have been observed. For instance, AtNRAMP2, located in Trans Golgi Network (TGN)/Early Endosomes (EE), is required for Mn supply of chloroplasts (Alejandro et al., 2017), which was shown before to also depend on Mn release from the vacuole by AtNRAMP3 and 4 (Lanquar et al., 2010). Accordingly, triple mutant analyses indicated that AtNRAMP2 and AtNRAMP3/4 operate successively. The mechanism that links AtNRAMP2/3/4 activity in the vascular system with chloroplast supply in mesophyll cells is unclear. A further player, the *trans*-Golgi-localised P_2A_-type ATPase *AtECA3*, is expressed exclusively in root and shoot vasculature and is required to maintain growth under low Mn supply by an unknown mechanism (Li et al., 2008); growth defects of *nramp2* and *eca3* mutants were additive (Farthing et al., 2023).

Abolition of *AtNRAMP6*, located in plasma membrane and secretory pathway in an Mn-dependent manner, similarly led to reduced Mn translocation to young leaves and reduced root growth in Mn-deficient conditions (Li et al., 2022). However, for unknown reasons, this defect became only apparent if *AtNRAMP1* was also knocked out. Moreover, grafting experiments indicated that *AtNRAMP1* and *6* were required in both root and shoot for the maintenance of root growth and the translocation of Mn, which might indicate shoot-to-root signalling of Mn status involving *AtNRAMP6* (Li et al., 2022).

An AtNRAMP2 homolog has recently been characterised in *Zea mays* (maize). In contrast to AtNRAMP2, ZmNRAMP2 is targeted to the tonoplast of xylem parenchyma cells and is required for root-to-shoot transfer of Mn (Guo et al., 2022). Knockout plants showed decreased Mn concentration in the xylem sap with a concomitant decrease in photosynthesis, suggesting a role of ZmNRAMP2 in Mn translocation from roots to shoots by release of vacuolar Mn into xylem parenchyma cells.

The expression of a diverse set of Mn transporters predominantly or exclusively in the vasculature of roots and shoots indicates the importance of vascular tissues as a site of storage and remobilisation and probably in sensing Mn status. It will be highly informative to identify the specific vascular cell types in which individual transporters are expressed in order to elucidate the mechanisms and pathways underlying vascular Mn homeostasis. Based on its subcellular localisation, it may be hypothesised that AtNRAMP6 facilitates Mn uptake into the Arabidopsis vasculature. Under conditions of Mn deficiency, redistribution may occur through the release from vacuoles by AtNRAMP3/4, export into the Golgi by AtECA3, and further transport into the TGN/EE via vesicular trafficking, before distribution from the TGN/EE via AtNRAMP2, with YSL transporters subsequently mediating the transfer into mesophyll cells.

## 5. Organellar Mn homeostasis

### 5.1. The vacuole – Mn pantry and dump

On a cellular level, the vacuole, as the plant’s by far largest compartment, may contain high amounts of Mn. Vacuolar exporters, such as AtNRAMP3/4 in Arabidopsis (Lanquar et al., 2010) or ZmNRAMP2 in maize (Guo et al., 2022), mediate the supply of Mn-demanding cells and organelles by Mn release from the vacuole. Mutant analyses indicate that interim storage in the vacuole is the default pathway, and that vacuolar release is thus essential to maintain optimum plant growth under limiting Mn availability (Lanquar et al., 2010). In poplar, a duplication of the *NRAMP3* ortholog was identified recently, of which only one functions as a vacuolar Mn/Fe exporter like in Arabidopsis. The second one was localised to the secretory pathway and involved in intercellular translocation in the vascular system, thereby altering plant-wide Mn homeostasis (Pottier et al., 2022).

Vacuolar Mn sequestration by AtMTP8 is employed to stock Mn in developing embryos of Arabidopsis (Chu et al., 2017; Eroglu et al., 2017). The transporter mediates the storage of the metal in subepidermal cells at the abaxial side of cotyledons and in cortical cells of the hypocotyl, which is required for efficient germination if the Mn supply of the mother plant is limited (Eroglu et al., 2017). Knockout of *AtMTP8* causes a reallocation of Mn to provascular strands, mediated by the vacuolar Fe/Mn transporter AtVIT1 (Kim et al., 2006). Intriguingly, the ubiquitous overexpression of *AtMTP8* did not change the tissue distribution of Mn within the embryo, indicating that this pattern is determined by either post-translational regulation mechanisms or upstream processes that need yet to be resolved (Höller et al., 2022).

Vacuolar sequestration also prevents Mn from interfering with critical processes upon Mn overload. Plants employing an acidification/reduction-based strategy of Fe mobilisation face the problem of excessive Mn uptake, since alongside Fe, Mn becomes more available in the acidified rhizosphere, and Mn is an alternative substrate of the Fe uptake transporter IRT1. Under these conditions, in Arabidopsis, expression of *AtMTP8* is strongly induced in cells expressing *AtIRT1*. Knock-out of *AtMTP8* renders plants hypersensitive to Fe-deficiency-induced chlorosis (Chu et al., 2017; Eroglu et al., 2016; Giehl et al., 2023). This vacuolar sequestration of Mn is required for the functioning of the Fe acquisition machinery, with ferric chelate reductase likely to be affected by elevated cytosolic Mn (Eroglu et al., 2016).

Intriguingly, in *Beta vulgaris* ssp. *vulgaris* (sugar beet) Fe deficiency did not induce the expression of any Mn-sequestrating *BvMTP* and provoked a lower Mn accumulation in roots compared to Arabidopsis, indicating functional differences in their Fe acquisition machinery (Alejandro et al., 2023). In *Lupinus albus* (white lupin), which accumulates massive amounts of Mn due to its P-mobilising activity, the role of MTP8-type transporters has been extended to Mn sequestration in leaves (Olt et al., 2022). Similarly, in rice as a monocot able to grow on submerged soils with very high Mn availability, mechanisms to cope with high Mn loads involve vacuolar sequestration in the shoot by MTP8-type transporters (Chen et al., 2013).

### 5.2. The chloroplast – Mn driving photosynthesis

The majority of Mn in the chloroplast is bound to PSII, where it is incorporated in the Mn_4_CaO_5_ cluster of the OEC, accepting electrons from H_2_O. On the luminal side of PSII, three extrinsic proteins (PsbO, PsbP and PsbQ) protect the OEC (Schmidt et al., 2016). Deficiency of Mn causes the dissociation of PsbP and PsbQ, leading to Mn release into the thylakoid lumen and ROS production, damaging light-harvesting pigments of PSII (Lilay et al., 2024). Mn-deficient plants of *Marchantia polymorpha*, an emerging model to study metal homeostasis, also showed increased non-photochemical quenching (NPQ) by increased cyclic electron flow to protect PSII against photoinhibition. Additionally, chloroplast ultrastructure was altered under Mn deficiency, reflected by disorganised thylakoids (Messant et al., 2023).

Mn supply of chloroplasts is conferred by transporters that were grouped in the uncharacterized protein family 0016 (UPF0016). In plants, this family has been assigned various names, including photosynthesis-affected mutant 71 (PAM71), PAM71-homolog (PAM71-HL), chloroplast manganese transporter (CMT), chloroplast-localised Ca^2+^/H^+^ antiporter (CCHA), photosynthesis-affected mutant 71-Like (PML) and bivalent cation transporter (BICAT). In this review, we employ the latter terminology, as it embraces all family members and reflects their function (He et al., 2021). Proteins of the BICAT family are related to Gcr1-dependent translation factor 1 (GDT1) in *Saccharomyces cerevisiae*, which was initially identified as a Ca^2+^ transporter localised in the Golgi (Demaegd et al., 2013). Later work demonstrated it also transports Mn (Thines et al., 2018). This dual functionality was also observed for its human homolog, TMEM165 (Stribny et al., 2020). In Arabidopsis, AtBICAT2/PAM71-HL/CMT1 translocates Mn across the inner envelope membrane, and mutants display symptoms that resemble Mn deficiency, including decreased photosynthetic efficiency and altered chloroplast ultrastructure (Eisenhut et al., 2018; Zhang et al., 2018). Moreover, in addition to Mn, AtBICAT2/PAM71-HL/CMT1 transports Ca^2+^ like its yeast and human counterparts, and is required for Ca^2+^ elevations in the chloroplast stroma induced by the onset of darkness (Frank et al., 2019). From the stroma, Mn is further translocated into the lumen by AtBICAT1/PAM71 localised in the thylakoid membrane (Schneider et al., 2016). Hence, the supply of the OEC with Mn is hampered in *bicat1/pam71* mutants. Complementation of a *bicat1/pam71* mutant demonstrated its functional similarity to human TMEM165 as well as the cyanobacterial Mn exporter MNX (Hoecker et al., 2021). Intriguingly, AtBICAT1/PAM71 also determines Ca^2+^ homeostasis in the chloroplast stroma, with its deletion augmenting the darkness-induced [Ca^2+^]_stroma_ transient (Frank et al., 2019).

While constitutive Mn supply of the thylakoid lumen is essential to drive the photosynthetic light reaction, and, as recent work showed, Mn activates protein kinases in the stroma (Espinoza-Corral et al., 2023), Ca^2+^ regulates various other processes in the chloroplast, including carbon assimilation and protein import (He et al., 2021). The requirement of uncoupling Ca^2+^ and Mn homeostasis raises the question, if and how selectivity of BICAT proteins is accomplished *in planta*. Unfortunately, neither free Mn ([Mn]) levels in chloroplast and cytosol nor the kinetics and selectivity of BICAT1/2 are known. Yeast GDT1 heterologously expressed in *Lactococcus lactis* has a *K_m_* of 15 and 83 μM for Ca^2+^ and Mn, respectively (Thines et al., 2018), while TMEM165 transports Ca^2+^ and Mn with a *K_m_* of 21 and 170 μM, respectively (Stribny et al., 2020). If plant BICATs operate in a similar concentration range, their *K_m_* for Ca^2+^ would be around 100-fold higher than steady-state [Ca^2+^]_cyt_. Despite a lower affinity for Mn than for Ca^2+^, such a transporter may still preferentially transport Mn when free Mn exceeds free Ca^2+^.

Transporters of the GDT1 family presumably function as H^+^ antiporters, and the activity of GDT1 and TMEM165 has been demonstrated to alter organellar pH homeostasis in yeast and human cells (Demaegd et al., 2013; Deschamps et al., 2023; Wang et al., 2020). Similarly, BICATs may thus impact pH in chloroplast compartments, which is relevant because the proton motive force across the thylakoid membrane drives ATP synthesis, and luminal pH regulates NPQ. Indeed, electrochromic shift measurements indicated an altered pH gradient in *bicat1/pam71* mutants (Schneider et al., 2016). In this respect, it is notable that the H^+^ antiport activity of yeast GDT1, when expressed in *Lactococcus lactis* is reversible (Deschamps et al., 2023). There are hints from yeast complementation experiments that this is also the case for plant BICATs: Besides complementing yeast mutants defective in Ca^2+^ and Mn efflux into organelles (Eisenhut et al., 2018; Frank et al., 2019; He et al., 2022; Schneider et al., 2016; Wang et al., 2016), BICATs also restore Mn influx in a yeast mutant devoid of the Mn uptake transporter Smf1 (Xu et al., 2023; Zhang et al., 2018; Zhang, Fu et al., 2021). Hence, the BICAT proteins are likely capable of mediating transport in both directions, as governed by the electrochemical potential gradient of the transported ions.

### 5.3. The secretory pathway – an emerging Mn hub

The secretory pathway links diverse cellular organelles and membranes by vesicular trafficking and fusion events. These processes may thus allocate Mn contained in vesicles to other compartments or the apoplast for utilisation or detoxification of the metal. Furthermore, numerous enzymatic processes within compartments of the secretory pathway are Mn-dependent. The absence of the P_2A_-type ATPase AtECA3, located in the Golgi apparatus of Arabidopsis, causes growth defects under low Mn supply (Farthing et al., 2023; Mills et al., 2008) and also renders plants sensitive to high levels of Mn (Li et al., 2008). The mechanistic basis of these phenotypes is unclear. Apart from ECA3, the Golgi apparatus of plants, like that of yeast and humans, contains proteins of the BICAT family. In Arabidopsis, AtBICAT3/PML3 has been shown to supply Mn for enzymatic activities in the *trans*-Golgi and to be localised primarily in these cisternae (He et al., 2022). Import of Mn into Golgi cisternae is likely energised by the H^+^ gradient, based on a slightly more acidic pH by 0.5 units in the Golgi as compared to the cytosol (Shen et al., 2013). Knockout of the ubiquitously expressed *AtBICAT3/PML3* impairs cell expansion and thus reduces plant growth (He et al., 2022; Yang et al., 2021). This was linked to an aberrant synthesis of cell wall matrix polysaccharides, in particular a severely decreased abundance of galactose moieties and 4-Gal linkages, which points to an affected β-1,4-galactan side chain substitution of rhamnogalacturonan I (He et al., 2022). The glycosyltransferases mediating this reaction, AtGALS1 to 3, are localised in the *trans*-Golgi and Mn-dependent (He et al., 2021). This specific glycosylation defect suggests that Mn supply of the Golgi may be cisternae-specific, with AtBICAT3/PML3 supplying the *trans* subcompartment. However, this model is challenged by another study that claimed AtBICAT3/PML3 to be present primarily in the *cis*-Golgi and *bicat3/pml3* mutants to be altered in protein glycosylation, leading to defects in cellulose synthesis and to display severe root tip degeneration under Mn deficiency (Yang et al., 2021). In addition, that study reported a hypersensitivity of *bicat3/pml3* mutants to Mn toxicity, unlike the findings of He et al. (2022). The discrepancies between the two studies need to be resolved, and may hint at a dynamic regulation of AtBICAT3/PML3 localisation and function.

An ortholog of BICAT3/PML3 has been characterised in rice (Xu et al., 2023). The consequences of a disrupted Mn supply of the Golgi in mutants for this transporter differ from those in Arabidopsis, with a major effect on hemicellulose biosynthesis. This is expected, as the cell wall composition of monocots differs from that of dicots.

A further role of AtBICAT3/PML3 was evident from the decreased seed setting of the mutants, caused by a male gametophyte defect (He et al., 2022; Zhang, Zhang et al., 2021). Tip-growing pollen tubes of *bicat3/pml3* showed abnormal growth, which went along with a severely diminished deposition of partially methyl-esterified homogalacturonan (HG) in their cell walls (He et al., 2022). HG is synthesised in the Golgi by proteins of the galacturonosyltransferase family. These harbour the DxD Mn-binding motif and are absolutely dependent on Mn (Amos et al., 2018).

Intriguingly, chloroplastic Mn concentrations of Mn-deficient plants were increased in *bicat3/pml3* mutants compared to the wild type, accompanied by a concomitant increase in photosynthetic activity (He et al., 2022). This alteration may be explained by competition between Golgi and chloroplasts for Mn, indicating that the activity of AtBICAT3/PML3 prevents Mn to be allocated to other compartments. It remains to be studied if this effect is harnessed by the plant to efficiently allocate the metal according to subcellular requirements. On the tissue level, Mn accumulation was also altered in *bicat3/pml3*. The mutant showed an increased Mn translocation to the shoot, interestingly caused by its absence in the shoot, as determined in reciprocal grafting experiments (He et al., 2022). This unexpected finding led to the hypothesis that Golgi Mn homeostasis may be involved in the regulation of Mn transport at the plasma membrane (Wege, 2022).

Efflux transporters or channels for Mn have not been identified in the Golgi yet. The metal may thus be translocated by vesicular trafficking, potentially followed by exocytosis, as suggested for Mn exclusion involving the Golgi-localised Mn transporter AtMTP11 (Peiter et al., 2007). Recently, relocalisation of AtMTP11 but not AtBICAT3/PML3 to the plasma membrane has been demonstrated upon inhibition of clathrin-mediated endocytosis, which supports an MTP11-dependent mechanism of vesicular Mn export by exocytosis (Vetal et al., 2025). In addition, Mn may be released from the Golgi by yet unidentified transport mechanisms.

The TGN/EE-localised transporter AtNRAMP2 is required for Mn supply of chloroplasts, albeit it is primarily expressed in the vasculature (Alejandro et al., 2017). As suggested by Kosuth, Leskova, Castaings et al. (2023), the interplay of AtNRAMP2 with AtECA3, AtBICAT3/PML3 and AtMTP11 may determine the cellular fate of Mn, whereby the release of Mn from the secretory pathway by AtNRAMP2 prevents its further traffic and replenishes cytosolic Mn. The testing of this attractive model requires the identification of the missing players and their functional interactions, as well as new methods to quantify Mn levels with subcellular resolution.

## 6. Distribution control – the multilayered regulation of Mn transport

### 6.1. Transcriptional regulation of Mn transport

The highly dynamic availability of Mn in the soil and the differing Mn demand and sensitivity of tissues require the regulation of Mn transport activities. However, the restriction of Mn uptake under overload conditions is limited, largely due to the low selectivity of the transport systems. For example, the expression of *AtIRT1*, which imports Mn alongside Fe, is under Fe-dependent transcriptional control and is even increased by excessive Mn availability. Increased activity of AtIRT1 under Fe deficiency requires Mn detoxification, which in Arabidopsis is conferred by AtMTP8 sequestering Mn in the vacuole (Eroglu et al., 2016). Massive transcriptional upregulation of *AtMTP8* is thereby controlled by FER-like iron deficiency-induced transcription factor (AtFIT), a central regulator of Fe deficiency-induced genes. Additionally, *AtMTP8* is under developmental control in embryos of developing seeds and becomes activated at late developmental stages (Eroglu et al., 2017). Apart from its regulation by Fe and seed developmental status, direct transcriptional regulation of *AtMTP8* by Mn has not been described. This is also true for many other Mn transporter genes, including *AtMTP11* (Delhaize et al., 2007), *AtBICAT1/PAM71* (Eisenhut et al., 2018), or *AtBICAT3/PML3* (He et al., 2022), which are unaffected by Mn availability. In contrast, *AtBICAT2/PAM71-HL/CMT1* is mildly downregulated by high Mn availability, which may serve to protect the chloroplast from Mn overload (Eisenhut et al., 2018). Contrarily, Mn deficiency provokes a slight upregulation of *AtNRAMP1* and *AtNRAMP2* (Alejandro et al., 2017; Cailliatte et al., 2010). However, directly Mn-responsive transcriptional networks and Mn sensors are unknown so far.

### 6.2. Post-transcriptional regulation of Mn transport

**Figure 2.**
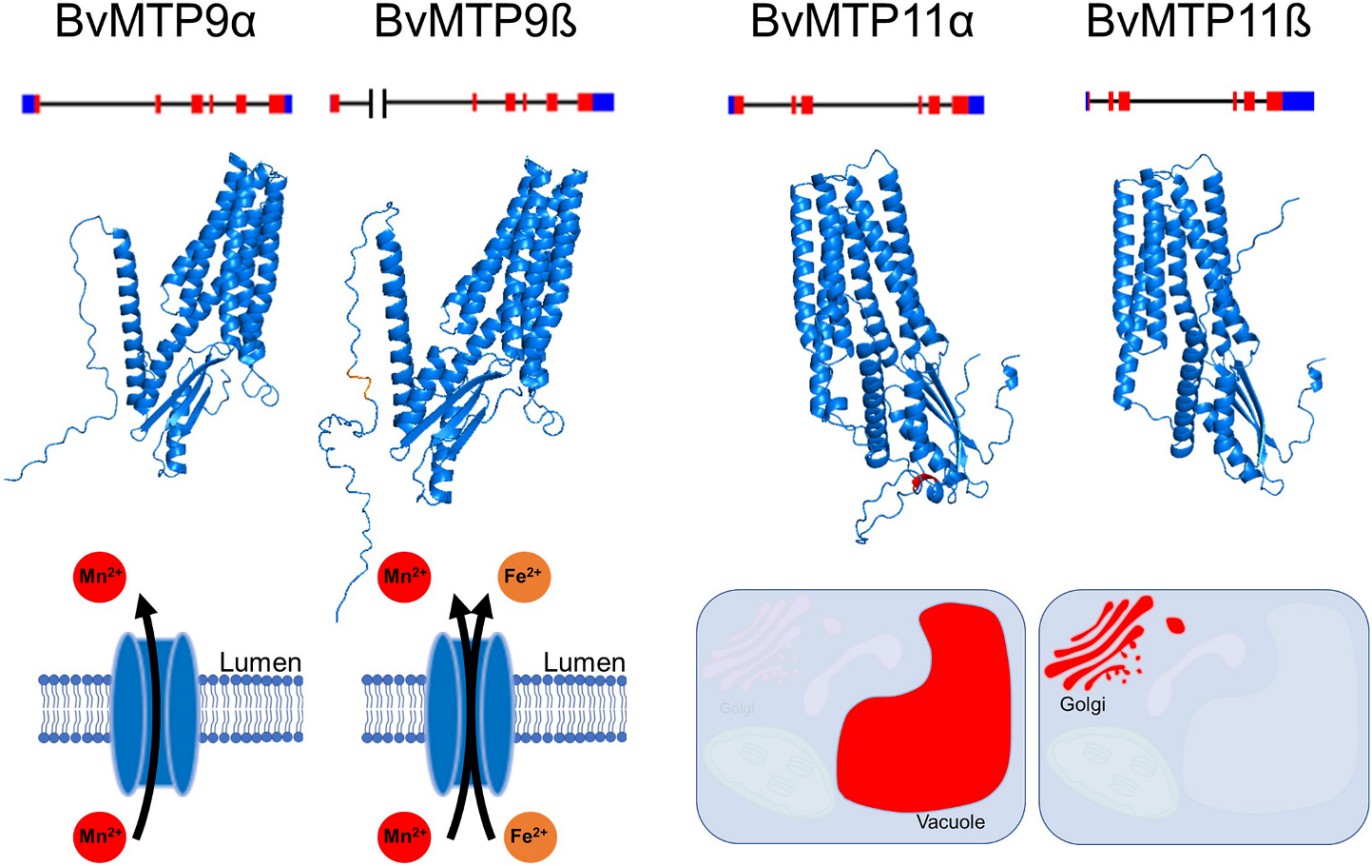
Alternative splicing of *BvMTP9* and *BvMTP11* from sugar beet. The blue boxes, red boxes and black lines represent untranslated regions, exons and introns, respectively. The additional 23 amino acids in the N-terminus of BvMTP9ß contain a D/ExxD/E (DITE) motif (orange area), which enables BvMTP9ß to transport Fe next to Mn. An N-terminal dileucine residue (red area) in BvMTP11α results in its targeting the vacuole, whereas BvMTP11ß localises to the secretory pathway similar to Arabidopsis AtMTP11. Data on protein structures were obtained using alphafold3 (https://alphafoldserver.com/, Abramson et al. (2024)), and models were created using PyMol (https://www.schrodinger.com/platform/products/pymol/).

There is increasing evidence that, as with animals, alternative pre-mRNA splicing also plays an important role in diversifying the properties of proteins encoded by individual genes in plants. In rice, the alternative splicing of a large number of genes was found to be provoked by the deficiency of nutrients, including Mn (Dong et al., 2018). Information on the role of this regulatory layer in Mn handling is limited. In Arabidopsis, the *AtNRAMP6* gene has been shown to express a partially spliced variant, which, however, appears to be truncated and non-functional (Cailliatte et al., 2009). In contrast, work on sugar beet demonstrated functional alterations of Mn transporters by differential pre-mRNA processing (Alejandro et al., 2023) (Figure 2). Sugar beet produces two splice variants of *BvMTP11*, *BvMTP11α* and *ß*, with distinct localisation. BvMTP11ß localised to the Golgi, as described before for AtMTP11, whereas BvMTP11α was targeted to the vacuolar membrane. Both variants, which show different expression patterns, complement an Mn-sensitive yeast mutant and the Arabidopsis *Atmtp11* mutant under excess Mn (Alejandro et al., 2023). Alternative splicing may thus serve to direct intracellular Mn fluxes to different compartments, and even to alter the Mn distribution within the plant. In addition to a change in protein localisation, alternative splicing was shown to modulate the substrate spectrum of another Mn transporter in sugar beet. Sugar beet BvMTP9, the role of which is still unknown also in Arabidopsis, exhibits two splice variants, *BvMTP9α* and *BvMTP9ß*. The N-terminus of BvMTP9ß contains an additional 23 amino acids, which confer it with the ability to restore growth of an Fe-sensitive yeast strain on high-Fe medium, next to the complementation of an Mn-sensitive yeast, as found for BvMTP9α (Alejandro et al., 2023). The N-terminus of BvMTP9ß contains a D/ExxD/E motif identified before to enable Fe transport by Mn-MTPs (Chu et al., 2017). The functional relevance of the regulation of selectivity or localisation by alternative splicing is unknown so far but potentially very significant.

### 6.3. Post-translational regulation of Mn transporter localisation

**Figure 3.**
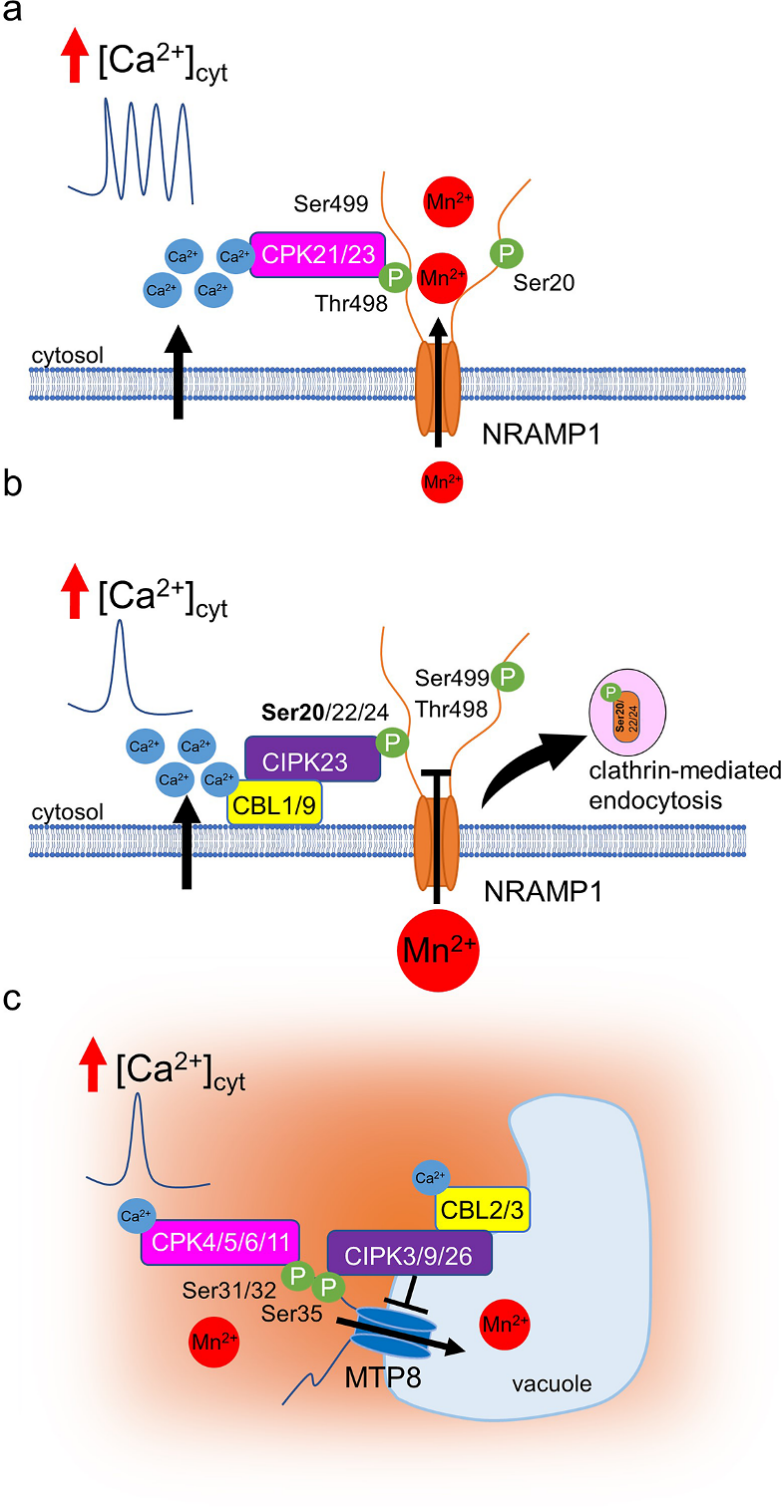
Schematic representation of phosphorylation processes in response to varying Mn levels. a. Under Mn deficiency, a delayed increase in [Ca^2+^]_cyt_ occurs with oscillatory kinetics, likely activating AtCPK21; AtCPK23 is constitutively active. These kinases phosphorylate the Thr498 residue of AtNRAMP1, resulting in its activation and subsequent Mn uptake. b. In conditions of Mn excess, a rapid and transient elevation in [Ca^2+^]_cyt_ activates the AtCBL1/9-AtCIPK23 module, leading to the phosphorylation of Ser20/22/24 of AtNRAMP1. This may trigger clathrin-mediated endocytosis of AtNRAMP1, thereby preventing further Mn uptake. c. Additionally, elevated [Ca^2+^]_cyt_ activates AtCPK4/5/6/11, which phosphorylates Ser31/32 of AtMTP8, promoting Mn export into the vacuole. Subsequently, the AtCBL2/3-AtCIPK3/9/26 module phosphorylates Ser35, resulting in the deactivation of AtMTP8.

The subcellular localisation determines the physiological role of a transport protein. Trafficking of Mn transporters to and from the plasma membrane may thus regulate the Mn influx. Indeed, AtNRAMP1, the principal high-affinity uptake transporter, has been shown to cycle between the plasma membrane and endosomes (Castaings et al., 2021). Localisation in the late endosome/multivesicular body compartment thereby depended on phosphatidylinositol 3-phosphate (PI3P) binding to the pleckstrin homology domain protein AtPH1 that localises in these vesicles (Agorio et al., 2017). The absence of AtPH1 caused an accumulation of AtNRAMP1 at the vacuolar membrane. Another study indicated an involvement of choline in the plasma membrane insertion of AtNRAMP1 by regulating endocytosis, possibly through phospholipase D activity (Gao et al., 2017). Potentially toxic levels of Mn evoke the internalisation of AtNRAMP1 by clathrin-mediated endocytosis (Castaings et al., 2021) (Figure 3). This response is dependent on the phosphorylation of Ser20 in the protein’s N-terminus. Phospho-dead mutations of this residue render AtNRAMP1 unable to undergo internalisation, and plants carrying this variant are hypersensitive to high Mn concentrations (Castaings et al., 2021).

Two families of protein kinases play a principal role in the phosphorylation of transport proteins: calcium-dependent protein kinases (CPKs) and CBL-interacting protein kinases (CIPKs). AtCIPK23 was identified to phosphorylate several target sites in the N- and C-termini of AtNRAMP1, including Ser20 (Kosuth, Leskova, Ródenas et al., 2023; Zhang et al., 2023). One study indicated that high Mn-induced AtNRAMP1 internalisation was abolished in an *Atcipk23* mutant, corresponding to Mn hypersensitivity of *Atcipk23* (Zhang et al., 2023). Phosphorylation of AtNRAMP1 by AtCIPK23 and the Mn hypersensitivity of an *Atcipk23* mutant were also shown in another work (Kosuth, Leskova, Ródenas et al., 2023). However, in this case, the *AtCIPK23* knockout did not have an effect on AtNRAMP1 internalisation, indicating a more complex regulatory mechanism, which is not completely unexpected. First, AtCIPK23 regulates a plethora of ion transporters, including AtIRT1 that also transports Mn. Second, the Ser20 site has been shown before to be also phosphorylated by AtCPK21 and 23 without promoting internalisation (Fu et al., 2022), indicating that multiple signalling pathways may converge at this point. The regulation of AtNRAMP1 internalisation demands further attention.

Permeation of Mn by the primary Fe uptake transporter AtIRT1 may lead to excessive Mn accumulation. Similar to AtNRAMP1, the protein cycles between the plasma membrane and TGN/EE vesicles, thereby undergoing clathrin-mediated endocytosis and monoubiquitination (Barberon et al., 2011). The internalisation is dependent on PI3P and mediated by non-Fe substrates (Barberon et al., 2014). Sensing of these ‘secondary’ metals by a histidine-rich domain triggers phosphorylation by AtCIPK23 and subsequent polyubiquitination by AtIDF1 to promote vacuolar targeting (Dubeaux et al., 2018). AtIRT1 thus functions as a transceptor, both transporting and directly sensing non-Fe metal cations to avoid their overaccumulation (Cointry & Vert, 2019). The affinity of the metal-sensing motif in IRT1 varies for different metals, being lower for Cd compared to Zn or Mn (Spielmann et al., 2022).

### 6.4. Post-translational regulation of Mn transporter function

Apart from regulating the trafficking of transporters, post-translational modifications may regulate their function. AtNRAMP1 is targeted by both CPKs and CIPKs, whereby phosphorylation by AtCPK21 and AtCPK23 promotes its activity by a yet unknown mechanism (Fu et al., 2022) (Figure 3). Both kinases phosphorylate Thr498, in addition to Ser20 and Ser22. Mutation of *AtCPK21* and *AtCPK23*, as well as their target Thr498, render the plant hypersensitive to low Mn availability, and the phospho-dead *nramp1^T498A^* mutant is affected in Mn transport, as determined by yeast complementation (Fu et al., 2022). As described above, Ser20 is also targeted by AtCIPK23, which may trigger its internalisation under certain conditions (Kosuth, Leskova, Ródenas et al., 2023; Zhang et al., 2023). In addition, Ser499 is phosphorylated by AtCIPK23 (Kosuth, Leskova, Ródenas et al., 2023), and the AtCPK21/23-targeted Thr498 might be as well (Zhang et al., 2023). Further work is necessary to disentangle potential interactions of the phosphorylation sites and/or the kinases and to resolve the apparently conflicting outcomes of different studies.

In addition to AtNRAMP1, the vacuolar Mn transporter AtMTP8 has been reported to be under post-translational control (Figure 3). Phosphorylation in its N-terminal domain by four CPKs promotes its activity, and mutation of the phosphosites or knockout of these *CPK* genes renders the plants Mn-hypersensitive (Zhang, Fu et al., 2021). As described above, AtMTP8 plays a role specifically under Fe deficiency and in developing seeds, and it is only expressed under these conditions (Eroglu et al., 2016; Eroglu et al., 2017). Intriguingly, its function under those circumstances was absolutely dependent on the phosphorylation of Ser31 and Ser32 by AtCPK4, 5, 6 and/or 11 (Zhang, Fu et al., 2021). It remains to be determined whether individual CPKs operate in specific tissues expressing *AtMTP8*. Phospho-mimetic mutation of AtMTP8 caused an overaccumulation of Mn in root vacuoles similar to a several thousand-fold overexpression (Eroglu et al., 2016; Zhang, Fu et al., 2021).

AtMTP8 is furthermore a target of AtCIPK3, 9 and 26, which phosphorylate it primarily at Ser35 (Ju et al., 2022). This modification negatively regulates its activity, and the deletion of the CIPKs or their interacting calcineurin B-like protein 2 (AtCBL2) and 3 renders plants more Mn-tolerant. Similar to the regulation by CPKs, the antagonistic regulation by CIPKs affected all MTP8-related phenotypes described so far, and its abolishment drastically increased Mn concentrations in root vacuoles (Ju et al., 2022). After exposure of plants to toxic Mn levels, phosphorylation by AtCPK5 and AtCIPK26 was induced successively, leading to the assumption that AtMTP8 activated by CPKs upon short-term Mn stress is again inactivated by CIPKs during long-term Mn exposure (Ju et al., 2022). It remains to be elucidated how such a mechanism is integrated into long-term housekeeping functions of AtMTP8, such as Mn loading of developing embryos.

### 6.5. Calcium signals – a new determinant of Mn homeostasis

Protein kinases of the CPK and CIPK families are activated by cytosolic free Ca^2+^ ([Ca^2+^]_cyt_), the former by direct binding of Ca^2+^ and the latter by interaction with Ca^2+^-binding CBL proteins. If Ca^2+^ is bound with a high affinity in the range of the steady-state concentration, kinase activity occurs without a further elevation of [Ca^2+^]_cyt_. This is the case for CPK23 that activates NRAMP1 (Fu et al., 2022) and may serve to unblock the transporter once it has reached its target membrane. Kinases binding Ca^2+^ with lower affinity require an elevation of [Ca^2+^]_cyt_ for activation, which was observed under both Mn deficiency and surplus. Depletion of Mn triggered unique, long-lasting, and very slow [Ca^2+^]_cyt_ oscillations in Arabidopsis roots (Fu et al., 2022), which initiated in the centre of the elongation zone after 2 hours of Mn depletion, and thereafter expanded to cell layers of the meristematic and differentiation zones. The oscillations continued for at least 6 hours at a frequency of 30 min (Fu et al., 2022). High-resolution imaging revealed that those Ca^2+^ elevations originated in cells of the epidermis, extended to cortical cells within 5 min, and continued with increasing intensity in the stele. These signals may be decoded by CPK21 to activate NRAMP1, which operates on the background of constitutive activation by CPK23.

High Mn availability also triggered a [Ca^2+^]_cyt_ elevation but with markedly different kinetics. Upon exposure to 1.5 mM Mn, a single increase was observed, either instantaneously or slowly rising within several minutes, as detected by aequorin luminescence and GCamP6f fluorescence, respectively (Fu et al., 2022; Zhang, Fu et al., 2021). The Ca^2+^ signal in response to excessive Mn was initiated in cells of the elongation zone, finding its maximum in the cortical cell layer. This Ca^2+^ elevation may be decoded by AtCBL1/9 that interacts with AtCIPK23, thereby inactivating and/or internalising AtNRAMP1 and AtIRT1 (Kosuth, Leskova, Ródenas et al., 2023; Zhang et al., 2023). Intriguingly, the AtCBL1/9-AtCIPK23 module also regulates K^+^ uptake by AtAKT1 (Xu et al., 2006) and NO_3_^−^ uptake by AtNRT1.1 (Ho et al., 2009). It is still a largely open question how the specificity of AtCIPK23 activity is conferred. In addition, AtCPK4/5/6/11 and the AtCBL2/3-AtCIPK3,9,26 module are likely activated by the high-Mn-triggered [Ca^2+^]_cyt_ transient to regulate AtMTP8 function (Ju et al., 2022; Zhang, Fu et al., 2021). However, as mentioned above, one role of AtMTP8 lies in the Mn loading of vacuoles in developing embryos, which likely demands a constitutive activity of AtMTP8 that is difficult to reconcile with Ca^2+^ signal-mediated activation.

The mechanisms generating low- and high-Mn-induced Ca^2+^ signals, as well as the Mn sensors upstream of those signals, are unknown.

## 7. Outlook

Over the last few years, we have seen a substantial progress in our understanding of Mn transport and its regulation in plants, in particular regarding the supply of organellar compartments and the post-transcriptional regulation of transporters. However, the co-operation of transport proteins operating in the same membrane or different organelles is still not well understood, albeit a picture is beginning to unfold, with the secretory pathway playing a central role. On the whole-plant level, the allocation of Mn and its handling in the vascular system is another field requiring attention, also with respect to the improvement of crop performance.

Our understanding of Mn homeostasis has primarily been derived from work on Arabidopsis in addition to rice, which is a monocot adapted to submerged soils with high Mn availability. The sketchy evidence available in a few other species already shows that there is a marked variability in Mn handling and that the toolbox of Mn transporters is used in a highly versatile manner, which calls for more extensive mechanistic studies in non-model species.

Our quantitative understanding of the feedback regulation of free and total Mn concentrations at different scales is still in its infancy. Advances in this area will require new methodological capabilities, such as Mn reporters, to assess the relevance, cooperation and regulation of transport proteins *in planta.* The design of specific reporters for Mn is challenging due to its low ligand affinity, but progress has recently been made in this area for a bacterial Mn reporter (Park et al., 2022). Finally, current discrepancies between studies from different laboratories may indicate a physiologically relevant adaptive capacity of the Mn-handling machinery, which can only be elucidated through a collaborative effort among the manganese community.

## Data Availability

No datasets were generated or analysed in this article.
