# Supplementary material for: Manganese handling in plants: Advances in the mechanistic and functional understanding of transport pathways
Source: Quant Plant Biol. 2025 Jun 20;6:e16. doi: 10.1017/qpb.2025.10012 (PMC12186567; doi:10.1017/qpb.2025.10012)
Supplement: Peer review history

# Editor report: Recommendation: Manganese handling in plants: Advances in the mechanistic and functional understanding of transport pathways — R1/PR2

**DOI:** 10.1017/qpb.2025.10012.pr2

**Affiliations:** CBSM - Faculty of Engineering, Universidad de Talca, Chile

Dear Edgar,

your revised manuscript has been seen again by one of the reviewers. The reviewer is satisfied with this revised MS as it stands. They further mention that your MS will make a major contribution to the literature on the way in which plants handle Mn. Thank you for the careful revision of the manuscript. And thanks again for your valuable contribution to the Research Topic “Quantitative approaches to cellular aspects of plant ion homeostasis”. It is highly appreciated.

Best regards, Ingo

# Editor report: Decision: Manganese handling in plants: Advances in the mechanistic and functional understanding of transport pathways — R1/PR3

**DOI:** 10.1017/qpb.2025.10012.pr3

**Affiliations:** RDP, ENS de Lyon, France

No accompanying comment.
